# Accuracy and Pitfalls in the Smartphone-Based Audiometry Examination

**DOI:** 10.22038/IJORL.2024.71187.3462

**Published:** 2024-03

**Authors:** Ramtry Waldi Berampu, Indri Adriztina, Ferryan Sofyan, Yetty Machrina, Ichwanul Adenin

**Affiliations:** 1 *Department of Otorhinolaryngology, Head and Neck Surgery* *, Faculty of Medicine* *, Universitas Sumatera Utara, Medan, Indonesia.*; 2 *Department of Physiology, Faculty of Medicine, Universitas Sumatera Utara, Medan, Indonesia.*; 3 *Faculty of Medicine, Universitas Sumatera Utara.*

**Keywords:** Audiometry, Smartphone, Hearing Loss, Hearing test, Smartphone-based

## Abstract

**Introduction:**

Approximately 466 million people suffer from hearing loss worldwide, with Indonesia ranking fourth in Southeast Asia. However, conventional pure-tone audiometry is not yet available in many areas because of its high cost. Numerous available smartphone-based audiometry applications are potential alternative screening tools for hearing loss, especially in Indonesia. This study examined the findings on the validation of smartphone-based audiometry applications to assess hearing functions available in Indonesia.

**Materials and Methods:**

Based on the established eligibility criteria, this study was conducted by browsing the relevant literature validating smartphone-based audiometry applications in Indonesia. Relevant study data, such as the author, year, location, implementation procedures, and outcomes, were extracted and summarized.

**Results:**

This systematic review found 17 relevant and eligible publications. Of the six applications tested, 5 were found to have good validity, such as uHear^TM^, Audiogram Mobile^TM^, AudCal^TM^, Hearing Test^TM^ e-audiologia, and Wulira^TM^. All smartphone-based audiometry was tested only for the air conduction threshold and was influenced by several factors.

**Conclusion:**

Because smartphone-based audiometry is inexpensive, simple, and more accessible than conventional audiometric testing, it can be useful as a screening modality or alternative approach to assess hearing function. Unfortunately, smartphone-based audiometry cannot replace conventional audiometry in diagnosing hearing impairment.

## Introduction

Hearing is the ability to recognize sounds (1) and is one of the human sensory systems necessary for humans to communicate with their surrounding environment at all times (2); however, hearing loss is commonly reported in every region and is estimated to affect 466 million people worldwide (5.5% of the world’s population), and this number is expected to rise to one in every four people by 2050 (3). 

After Sri Lanka, Myanmar, and India, Indonesia ranked fourth in Southeast Asia with the highest rate of hearing loss (4). 

The national prevalence rate of hearing loss in Indonesia is 2.6%, with East Nusa Tenggara Province leading the way with a rate of 3.7%, followed by Lampung in the second position with a rate of 3.6% (5). Currently, 34 million children have hearing loss, comprising 60% preventable cases (3). More than 1 billion young people aged 12–35 years are at risk of hearing loss due to recreational exposure to loud noises. Approximately one-third of people aged >65 years have hearing loss, with most cases occurring in the South Asian, Asia Pacific, and sub-Saharan African regions. Hearing loss has far-reaching consequences, can be very harmful, and can also lead to social isolation, with people becoming lonely and frustrated, especially among elderly people (3).Early detection (screening) and prompt treatment are important to avoid hearing loss, which has several negative consequences (6). A hearing function examination, using conventional pure-tone audiometry (PTA) as the gold standard for assessing hearing function, can be performed to determine whether someone has a hearing loss (7,8). However, although a conventional audiometer is a highly recommended test in hearing function tests because of its accuracy, the use of PTA is still not fully applicable because of substantial challenges in many areas, particularly in lower-middle-income areas (6). Because conventional audiometers are very expensive, these tools are still limited and not widely available in many areas. As a result, access to hearing function tests remains challenging, and the costs incurred by an individual for each examination are also quite high (6). Although the number of conventional audiometers is still limited and not widely available in many regions, various new, innovative, low cost, easy-to-use, and automated technologies have been developed to assess hearing function. One of these is smartphone-based audiometry, which can assess hearing functions. Smartphone application developers have used this technology to develop applications that perform independent hearing screening tests. 

With the growing use of smartphones globally, audiometry applications offer a promising avenue for screening hearing loss (6). Smartphone-based audiometric solutions have been proposed as a means of lowering costs and increasing access to hearing function tests (9,10). Other studies have found tangible evidence of the use of smartphone applications for hearing assessment in different populations (10-13). A randomized controlled trial conducted in Turkey has shown that the use of smartphone-based audiometric tests produces results comparable to that of conventional audiometry (14).Smartphone-based audiometry tests are also widely available in Indonesia and can measure hearing function; however, their scientific validity has not been thoroughly reviewed. Therefore, studies on the validation of smartphone-based audiometry available in Indonesia for assessing hearing function are required with smartphone-based audiometry compared to conventional audiometry’s gold standard. 

## Materials and Methods

This systematic review used the Preferred Reporting Items for Systematic Reviews and Meta-Analyses (PRISMA) to conduct the literature search. Based on the established eligibility, this study was conducted by browsing the relevant literature validating smartphone-based audiometry applications in Indonesia. Smartphone-based audiometry applications reviewed in the literature were checked for availability in Indonesia by searching for the application’s name on the most popular smartphone commercial application stores in Indonesia, namely, the Google Play Store (Android) and the Apple App Store (iOS).

*Literature Search Method*

Literature that validated smartphone-based audiometry applications was searched online using three databases, namely, PubMed, ScienceDirect, and Google Scholar, with the following keywords: (Accuracy OR Accurate OR Valid) AND (Audiometry OR Audiometer) AND (Smartphone OR Android OR iOS). This systematic review was conducted using PRISMA.

*Eligibility Criteria*

The included literature must meet the inclusion and exclusion criteria. The inclusion criteria are as follows:

- Participants: All study participants were aged over 4 years.
- Intervention: Smartphone-based PTA application in Indonesia.
- Comparison: Conventional PTA.
- Outcomes: Validity in measuring hearing function (study results in the form of application performance assessments such as sensitivity, specificity, positive and negative predictive value, positive/negative likelihood ratio, receiver operating curve analysis, kappa Cohen, Intraclass Correlation Coefficient, the difference in average hearing threshold, and Cronbach’s alpha).
- Study design: The primary study was conducted in 2011–2022.

This study excluded literature that could not be accessed in full text, literature in the form of reviews, and literature that did not use English.

*Data Analysis Method*

 The synthesis of qualitative data using a meta-aggregation approach was used as the data analysis method in this study. All data from the literature included in accordance with PRISMA were collected using data collection standards established by us. Relevant data of the study, such as the author, year, location, implementation procedures, and outcomes, were extracted and summarized.

## Results

**Fig 1:**
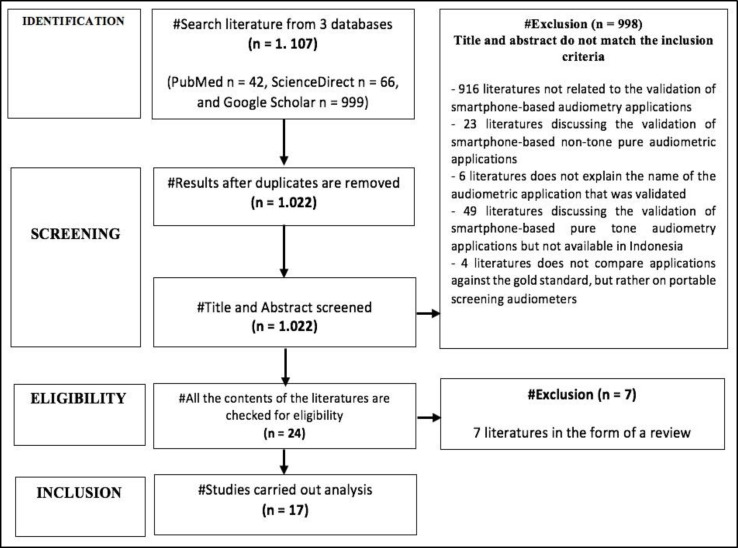
Results of the literature search with PRISMA stages

A total of 1,107 publications were identified by searching three literature databases: PubMed, ScienceDirect, and Google Scholar. The literature was then systematically examined using the PRISMA stage (Fig. 1), with 85 of them being excluded due to duplicates. 

The remaining titles and abstracts were reviewed for relevance, and 998 were determined not to meet the inclusion criteria. The relevant literature was reviewed for eligibility, and 17 papers were eventually selected to be included in this study (7,14-29). A total of 17 papers included in the collection discussed the validation of smartphone-based audiometry applications available in Indonesia. This systematic review tested six applications for validity in (Table 1). uHear^TM^, which has been validated by eight papers, is the most validated application for the iOS operating system. Other validated iOS applications include Audiogram Mobile^TM^, which was validated in two papers, and AudCal^TM^, which was validated in one paper. Not only iOS-based audiometry applications but also smartphone-based audiometry applications with an Android system were also found, including four papers validating the Hearing Test^TM^ e-audiologic application and one article validating each, the Hearing Test Pro^TM^ e-audiologic and Wulira^TM^ (7,14-29). All of the literature included in this survey was a primary study conducted over several years. In 2012, one research article was found; in 2015 and 2017, two papers were found. In 2016, 2018, and 2020, four publications were found. The research for the included publications was also conducted in different countries. Nigeria and Pakistan most commonly conduct validation research on audiometry applications, with several other countries, namely, South Africa, USA, Belgium, Israel, Canada, Malaysia, Oman, Poland, Spain, Taiwan, Turkey, New Zealand, and Uganda. All included literature was in the form of an experimental in-subject design with a prospective, cross-sectional study. The number of patients included in each study varied. The smallest sample size was 20 people, and the largest was 200 people, with a total sample size of 1,455 people drawn from the entire literature (2,910 ears). Almost 60% of the participants were males, ranging from 8 to 91 years (7,14-29).

As shown in Table 1, the uHear^TM^ application has generally good validity (7,15-18), with only three studies finding poor validity (17,19,20). Due to the noisy test environment, an uncelebrated tool, and a 16-bit digital-to-analog converter in iPod/iPhone devices, the uHear^TM^ application’s dynamic range is limited to approximately 85 (15–100) dB (19). Because elderly participants could not operate smartphones properly, the validity of uHear^TM^ deteriorated (17). The noisy test environment and difficulties understanding the application instruction language were the study’s limitations that resulted in poor validity of the obtained results (20). The uHear^TM^ application is beneficial for screening moderate to severe hearing loss (>40 dB) and is more accurate at higher frequencies (i.e., 2000, 4000, 6000, and 8,000 Hz frequencies). Conversely, the validity of low-frequency measurements was found to be poor (7,16,17,19,20). This is due to environmental noise and occlusion caused by poor transducers. Uncontrolled test environment noise and inefficient use of transducers cause sound in the test environment to enter the ear canal and interfere with pure-tone transmission, causing a decreased sound pressure of the pure-tone in the ear canal, which in turn vibrates the tympanic membrane and auditory bones. This is more contrasted at low frequencies (250–1,000 Hz) because the number of sound waves produced at low frequencies is less than that produced at high frequencies; therefore, low-frequency pressure in the ear canal significantly increases the hearing threshold because it vibrates less than at high frequencies. The validity of the uHear application has also been proven to improve when performed in a low-noise environment (7,15,19). 

**Table 1:** Smartphone-based audiometry validati

**Research**	**Frequency Test Used** **(Hz)**	**Tools and Transducers Used**	**Calibration Execution**	**Gold Standard / Reference**	**Definition of Hearing Loss Used Outcome Analysis**	**Results (Outcomes)**
Szudek et al(2012) Kanada	1000, 2000, 4000	Tool: iPod Transducer: Standard apple earbuds Test place: Under doctor/researcher supervision in a quiet room (ambient noise < 50 dBA) and soundproof cubicle	The user was in a very quiet environment with the transducer installed correctly, the device volume set to 50% and the type of device being used is inputted in the app. The test is conducted with a single combination of the device and earbuds to avoid equipment bias	Conventional pure tone audiometry was conducted by an audiologist in a soundproof booth	PTAv > 40 dB HL at 1.000 Hz, 2000 Hz and 4000 Hz in both ears	Quiet room: Sensitivity: 98% Specificity: 82% Soundproof booth: Sensitivity: 100% Specificity: 90%
Peer and Fagan (2015) South Africa	250, 500, 1000, 2000, 4000, 6000	Tool: iPhone 4 Transducer: Standard apple earbuds Test place: Audiometry was conducted under the supervision of doctors/researchers at 3 different test environment conditions	Not reported The test was conducted with a single combination of the device and headphones	Conventional pure-tone audiometry was performed by an audiologist in a soundproof booth	PTAv > 40 dB HL at 500 Hz, 1000 Hz, 2000 Hz and 4000 Hz in the better ear	Waiting room: Sensitivity: 100% Specificity: 64% Kappa Cohen: Low frequency (-0,0516)-0,5126 *(bad to moderate)* High frequency 0,339-0,517 *(medium)* Quiet room: Sensitivity: 100% Specificity: 74% Kappa Cohen: Low frequency (-0,0101)-0,5207 *(bad to moderate)* High frequency 0,73-0,79 *(moderate to good)* Soundproof booth: Sensitivity: 100% Specificity: 88% kappa Cohen: Low frequency 0,097-0,66 *(bad to good)* High frequency 0,75-0,94 *(moderate to very good)*
Al-Abri et al (2016) Oman	500, 1000, 2000, 4000	Tool: Apple iPad Transducer: Standard apple earbuds Test place: Participants were evaluated in clinical rooms (quiet rooms) and soundproof booths	Not performed	Conventional pure-tone audiometry was conducted by an audiologists in soundproofed booths	Mean hearing level difference between conventional audiometer and uHear^TM^ by paired t-test. Difference of 10 dB or more was considered significant.	Quiet room: Statistically significant differences between of uHear^TM^ and conventional pure tone audiometry results were found in the clinic at all frequencies. Soundproof booth: Significant difference was found between uHear^TM^ and conventional pure tone audiometry result in a soundproof booth at 500 Hz frequency
Livshitz et al (2016) Israel	500, 1000, 2000, 4000, 6000	Tool: Apple iPad Transducer: AKG K512 MK II circumaural headphones Test Place: The test was conducted in a quiet room	Not reported The test was conducted with a single combination of the device and headphones	Conventional pure-tone audiometry was conducted by an audiologist in a soundproof booth	PTAv > 35 dB HL at 500 Hz, 1000 Hz, 2000 Hz, 4000 Hz and 6000 Hz in the better ear Mean hearing level difference between conventional audiometer and uHear^TM^ in both ears (*p* < 0,05)	Statistically significant difference was found between uHear^TM^ and conventional pure-tone audiometry results across at all frequencies of both ears (paired t-test, *p* < 0,001) After the results were subtract a constant factor of 25 dB for each of the frequencies to compensate ambient noise : No significant mean hearing level difference was found between uHearTM and conventional pure tone audiometry at all frequencies of both ears (*p* > 0.05) Sensitivity: 76,5% Specificity: 90,7% PPV: 76,5%. NPV: 90,7%
Lycke et al (2016) Belgia	500, 1000, 2000, 4000, 6000	Tool: iPod Transducer: Standard iPod earbuds Test Place: The test was conducted by an audiologist in a quiet room.	Not performed	Conventional pure tone audiometry was conducted by an audiologist in a soundproof booth	PTAv > 40 dB HL at 500, 1000, and 2000 Hz in both ears Mean hearing level difference between conventional audiometer and uHear^TM^ in both ears (Wilcoxon Matched Pairs Signed Ranks Test, *p* < 0,001)	Sensitivity: 100% Specificity: 36,4% PPV: 22, 2% NPV: 100% Significant difference was found between uHear^TM^ and conventional pure tone audiometry results at the frequencies of 500 Hz, 1.000 Hz, 2.000 Hz (*p* < 0,001) No significant difference was found between uHear^TM^ and conventional pure tone audiometry results at 4.000 Hz frequency (*p* = 0,327) ROC: 0,98 ± 0,14 *(Very good)*
Ogah (2017) Nigeria	250, 500, 1000, 2000, 4000, 6000, 8000	Tool: iPhone Transducer: Headphones type was not reported Test place: Conducted by the patient under the supervision of a doctor/researcher in a quiet room	Not reported The test was conducted with a single combination of the device and headphones	Conventional pure tone audiometry was conducted by an audiologist in a soundproof booth	Hearing level > 40 dB in each ear Mean hearing level difference between conventional audiometer and the uHear^TM^ in both ears (Chi-test, *p* < 0,05) Significant difference was found between uHear^TM^ and conventional pure tone audiometry results at lower frequencies, namely 250 Hz, 500 Hz and 1000 Hz (*p* < 0,0001) No significant difference was found between uHear^TM^ and conventional pure tone audiometry results at higher frequencies (2000 Hz, 4000 Hz, 6000 Hz, 8000 Hz) with *p* value = 0,8914 (*p* > 0,05)	
Anuar et al(2018) Malaysia	250, 500, 1000, 2000, 4000, 8000	Tool: Apple iPad Transducer: Standard apple earbuds Test place: The test was conducted in the clinic	Not reported The test was conducted with a single combination of the device and headphones	Conventional pure tone audiometry was performed by an audiologist in a soundproof booth	PTAv > 40 dB HL at 250 Hz, 500 Hz, 1000 Hz, 2000 Hz, 4000 Hz, and 8000 Hz in both ears Mean hearing level difference between conventional audiometer and uHear^TM^ of both ears (paired t-test, *p* < 0,05) Sensitivity: 54% Specificity: 99% Kappa Cohen at frequency:	250Hz: 0,254 *(fair)* 500 Hz: 0,314 *(fair)* 1000 Hz: 0,303 *(fair)* 2000 Hz: 0,290 *(fair)* 4000 Hz: 0,291 *(fair)* 8000 Hz: 0,397 *(fair)* Significant mean hearing level difference was found between uHear^TM^ and conventional pure tone audiometry results (*p* < 0,0001)
Li et al (2020) Taiwan	250, 500, 1000, 2000, 4000, 6000	Tool: iPhone 4S Transducer: Sennheiser HD201 circumaural headphones Test place: The test was conducted by an audiologist in soundproof booth (average test environment noise level < 35 dB)	Not reported	Conventional pure tone audiometry was conducted by an audiologist in soundproofed booth	PTAv > 40 dB HL at 500 Hz, 1000 Hz, 2000 Hz, and 4000 Hz in the better ear.	Sensitivity: 92% Specificity: 76% Likelihood Ratio Positive: 3,80 Negative Likelihood Ratio: 0,11
Corry et al (2017) New Zealand	250, 500, 1000, 2000, 4000, 8000	Tool: Apple iPad Transducer: Standard apple earbuds Test place: Performed by an audiologist in a soundproof booth	Calibration was performed using a Sound Level Meter (SLM), which was in contact with one of the headphones. The application volume was set to 60 dB for 1 kHz. The headphone output of the app is then adjusted to 60 dB(A) on the SLM	Conventional pure-tone audiometry was performed by an audiologist in a soundproof booth	Mean hearing level difference between conventional audiometer and Audiogram Mobile^TM^ results (repeated-measures ANOVA)	Significant mean hearing level difference was found between the Mobile^TM^ Audiogram and conventional pure tone audiometry results (F(1, 19): 16,635, *p* < 0,001) No significant difference was found between test and retest results for standard conventional pure tone audiometry or application (indicating good retest-test reliability)
Kelly et al (2018) USA	250, 500, 1000, 2000, 4000, 6000, 8000	Tool: Apple iPad Transducer: Bose QuietComfort 15 acoustic noise-canceling headphones Test place: Conducted by the patient in the clinic waiting room (test environment noise level was 40-70 decibels) and quiet room	Not reported	Conventional pure tone audiometer was conducted by an audiologist in a soundproof booth	Hearing level > 20 dB at each frequency of each ear	Quiet room Sensitivity: 85,3% Specificity: 95,1% Waiting room Sensitivity: 87,6% Specificity: 92,3%
Larossa et al (2015) Spanyol	500, 1000, 2000, 3000, 4000, 8000	Tool: Various apple devices (iPhone 4, 5, 5c, 5s, iPad2) Transducer: Standard apple earbuds Test place: Test was performed by an audiologist in a quiet room	Calibrated by biological method (The sound outputted by AudCal^TM^ was analyzed using manual comparison between clinical audiometer and standard headphone and EarPod paired with iPhone)	Conventional pure tone audiometry was conducted by an audiologist in a soundproof booth	PTAv > 20 dB HL at 500 Hz, 1000 Hz, 2000 Hz, 4000 Hz of each ear	Kappa cohen: 0,894 *(very good)* ICC: 0,93 *(very good)* Significant mean hearing level difference: 0,21±6,38 dB Cronbach's Alpha: 0,96 *(very good)*
Renda et al(2016) Turki	250, 500, 1000, 2000, 4000, 6000, 8000	Tool: Samsung Galaxy GT-19500 S4 Transducer: Bundled headphones of an unreported type Test place: Test was performed by the patient (ambient noise < 39 dB based on SoundMeter™ app)	Not performed	Conventional pure tone audiometry was conducted by an audiologist in a soundproof booth	Mean hearing level difference between the conventional audiometer and the e-audiologia Hearing Test^TM^ in both ears (Wilcoxon Matched Pairs Signed Ranks Test, *p* < 0,05)	ICC: 0,878-0,933 *(very good)* Significant mean hearing level differences were found between Hearing Test^TM^ e-audiologia and conventional pure tone audiometry at the frequencies of 500 Hz, 1000 Hz, 2000 Hz, and 6000 Hz, (*p* < 0,05) No significant difference was found between Hearing Test^TM^ e-audiologia and conventional pure tone audiometry results at the frequencies of 250 Hz, 3000 Hz, 4000 Hz, and 8000 Hz, (*p* > 0.05)
Masalski et al(2018) Polandia	250, 500, 1.000, 2.000, 4.000, 6.000, 8.000	Tool: Android devices (Samsung, Huawei and Sony with various models) Transducer: Bundled Headphones of an unreported type Test place: Test was performed by the patient with an assist from an audiologist in a soundproof booth	Calibrated by biological method (The sound outputted by Hearing Test^TM^ was analyzed using manual comparison between clinical audiometer and headphone paired with device)	Conventional pure tone audiometer was conducted by an audiologist in a soundproof booth	Hearing level > 30 dB at any of the frequencies: 500 Hz, 1.000 Hz, 2.000 Hz, or hearing level > 25 dB at more than one frequency: 500 Hz, 1.000 Hz, 2000 Hz of each ear Hearing level > 50 dB at 4000 Hz of each ear Sensitivity: 98% Specificity: 79% ICC: 0,85 Cronbach's Alpha: 0,92 *(very good)*	
Asghar et al 2020) Pakistan	250, 500, 1000, 2000, 4000, 8000	Tool: Samsung Galaxy Note 4 Transducer: Bundled Headphones of an unreported type Test place: Test was performed by the patient in a quiet room	Not reported	Conventional pure tone audiometry was performed by an audiologist in a soundproof booth	Significant mean hearing level difference between conventional audiometer and Hearing Test^TM^ e-audiologia in both ears (Wilcoxon Matched pairs Signed Ranks Test, *p *≤ 0,05)	Significant mean hearing level difference between Hearing Test^TM^ e-audiologia and conventional pure tone audiometry was found with *p* < 0,05 ICC: 1000 Hz: 0,42-0,59 *(fair)* 250 Hz, 500 Hz, 2000 Hz: 0,60-0,74 *(good)* 4000 and 8000 Hz: 0,75-1,00 *(very good)*
Najeeb et al(2020) Pakistan	250, 500, 1000, 2000, 4000 8000	Tool: Samsung Galaxy Note 4 Transducer: Bundled Headphones of an unreported type Test place: Test was performed by the patient in a quiet room	Not reported	Conventional pure tone audiometry was performed by an audiologist in a soundproof booth	Mean hearing level difference between conventional audiometer and e-audiologia Hearing Test^TM^ in both ears (Wilcoxon Matched Pairs Signed Ranks Test, *p *≤ 0,05)	Significant mean hearing level difference was found between Hearing Test^TM^ e-audiologia and conventional pure tone audiometry (*p* < 0,05) ICC: 1000 Hz: 0,42-0,59 *(fair)* 250 Hz, 500 Hz, 2000 Hz: 0,60-0,74 *(good)* 4000 Hz and 8000 Hz: 0,75-1,00 *(very good)*
Aremu (2018) Nigeria	250, 500, 1.000, 2000, 4000, 6000, 8000	Tool: Android smartphone with unreported model Transducer: Not reported Test place: Test was performed by the patient in audiometric booth	Not reported	Conventional pure-tone audiometry was performed by an audiologist in a soundproof booth	PTAv > 40 dB at each frequency of each ear Mean hearing level difference between conventional audiometer and Hearing Test Pro^TM^ e-audiologia of both ears (Wilcoxon Matched Pairs Signed Ranks Test, *p* < 0.05)	At lower frequencies (250 Hz, 500 Hz, 1000 Hz): Sensitivity: 29,44% Specificity: 55,83% Significant mean hearing level difference was found between the Hearing Test Pro^TM^ e-audiologia and conventional pure tone audiometry (*p* < 0,001) At higher frequencies (2000 Hz, 4000 Hz, 6000 Hz, 8000 Hz): Sensitivity: 38,06% Specificity: 66,22% No significant mean difference was found between Hearing Test Pro^TM^ e-audiologia and conventional pure tone audiometry (*p* = 0,987, *p* > 0,05)
Batte et al(2020) Uganda	500, 1000, 2000, 3000, 4000	Tool: Android smartphone with unreported model Transducer: Earbuds Test place: The test was conducted by a radiologist (with audiometry training and 1-year of experience) in a soundproof booth.	No performed	Conventional pure tone audiometry was performed by trained radiologists in soundproof booths	Hearing level > 25 dB at each frequency of each ear	Right ear Sensitivity: 91,4% Specificity: 93,2% Left ear Sensitivity: 88,4% Specificity: 91,5%

Notes. TM: Trademark; Hz: Hertz, iOS: iPhone Operating System, PTAv: Pure Tone Average, PTA: Pure Tone Audiometry, dB: decibels, dBA: decibels (grade A), db HL: decibels Hearing Loss, PPV: Positive Predictive . Value, NPV: Negative Predictive Value; SLM: Sound Level Meter, ANOVA: Analysis of Variance, ICC: Intraclass Correlation Coefficient, ROC: Receiver Operating Characteristic

Audiogram Mobile^TM ^is another application included in this systematic review. This application is beneficial for screening mild to severe hearing impairment. The Audiogram Mobile^TM^ application has been validated in two publications (21,22). The Audiogram Mobile^TM^ application has good validity in screening for mild to severe hearing loss (>20 dB) when used in a low-noise environment. However, it has not achieved as much as uHear^TM^ because of noise background and cut-off threshold-based (22). 

Conversely, the Mobile^TM^ Audiogram had low validity but high reliability. Poor validity is due to the type of transducer used, which did not occlude the ear, leading to some tone leakage from the ear canal, resulting in a lower overall sound pressure level in the ear canal. Another reason for participant distraction during application testing was that the examiner was in a soundproof booth with the patient. When the tester is present, the participant may find it more distracting because they should pay attention to many factors, including the sound of the tester operating the iPad. Despite some limitations in its use, this application helps screen mild or more severe hearing impairment by considering factors affecting the measurement results. The drawback that limits the use of this application is that it is paid and only available on the Apple iPad (21).

The AudCal^TM^ application has excellent validity and reliability and is the last application validated with the iOS operating system. Only one study proved its validity and reliability. The average hearing threshold difference between AudCal^TM^ and conventional PTAs was 0.21 ± 6.38 dB; however, the study did not explain whether the difference was significant or not. The kappa Cohen analysis was used to analyze the validity of AudCal^TM^. The AudCal^TM^ kappa Cohen value was found to be very good, indicating that the hearing threshold match between the AudCal^TM^ application, and the conventional audiometer in assessing hearing function was perfect. The test and retest reliability of this application were excellent. In this study, the validity and reliability of the AudCal^TM^ application were excellent; therefore, it can be used as a screening tool to assess mild to severe hearing loss (>20 dB). However, further research is needed to increase the knowledge and confidence in its use (23).

Another application with a validated Android operating system is Hearing Test^TM^ e-audiology, which can be downloaded for free from the Google Play Store. The Hearing Test^TM^ e-audiology application can screen for mild hearing loss. Four studies that validated the e-audiologic Hearing Test^TM^ application found excellent compatibility with conventional audiometry in assessing hearing function. Although its consistency with standard audiometry in assessing hearing function revealed perfect validity, the Hearing Test^TM^ e-audiologic application still had a significant difference in the threshold obtained (14,24-26).

The Hearing Test^TM^ e-audiology application found remarkable differences at frequencies of 500, 1,000, 2000, and 6,000 Hz, but not at 250, 3,000, 4,000, and 8,000 Hz. This significant difference, particularly at 500 and 1,000 Hz frequencies, was suspected to be due to the noise around the test. No significant difference was observed at 250, 3,000, 4,000, and 8,000 Hz frequencies. However, the hearing threshold of smartphone-based audiometry was better than that of conventional audiometry in the right ear. This could be because users can drive it to any result they want due to self-administration (14). Other studies found a significant difference between Hearing Test^TM^ e-audiologia and PTA at frequencies of 250, 500, 1.000, 2.000, 4.000, and 8.000 Hz (25,26). This resulted from a noisy testing environment in a quiet room (25). In contrast to three previous studies conducted in quiet rooms, this application demonstrated excellent validity in a soundproof room. It not only had an excellent intraclass correlation coefficient (0.85) but also a good sensitivity and specificity (99% and 79%, respectively). The application’s consistency in assessing the patient’s hearing threshold (reliability of the instrument) is promising (Cronbach’s alpha value = 0.92), with a slight difference in the average test and retest threshold of 0.1 dB. Based on the explanation provided in the four publications above, the Hearing Test^TM^ e-audiology application can be used as a screening tool for mild to severe hearing loss (>25–50 dB) by considering the factors that influence the measurement results (24).

Hearing Test Pro^TM^ e-audiologia is another validated audiometry app for Android. In this systematic review, one study appropriately verified the eligibility of the Hearing Test Pro^TM^ e-audiologia application. They explained that the Hearing Test Pro^TM^ e-audiologia application has low validity and thus cannot be used as a screening tool for hearing loss. The drawback of this application is that it costs money to access, which limits its use. Its validity is determined by the type and installation of the transducer and calibration issues. Nonetheless, studies on this application are still minimal; thus, further studies are required to prove the application’s validity (27).

The Wulira^TM^ application is the final application on the validated Android system with good validity; however, due to a few limitations, the validity of the application was less than optimal. Its limitations were poor sound attenuation by the earbuds and a noisy testing environment. The excellent validity of the WuliraTM application and its similarity to conventional audiometry explain why it is suitable for use as a screening tool for mild hearing loss. However, this application was tested in a soundproof room and will undoubtedly be implemented independently by the user in a quiet (not soundproof) environment. Thus, further research is required to test its validity in different environmental conditions to increase the knowledge and evidence of its reliability (28).

## Discussion

Advances in smartphone technology have resulted in a plethora of healthcare applications, many of which can be self-administered by trained professionals and nonprofessionals to assess clinical populations and screen at risk individuals in areas where conventional audiometry is inaccessible. Many hearing test apps are free or inexpensive and are available to the public worldwide, including in Indonesia. They are easily accessible on smartphones running iOS (Apple) or Google Play (Android) operating systems. Self-administered hearing tests can be helpful for individuals who do not have access to auditory services or for monitoring their hearing status (19,23,29).

Research on the validity of smartphone-based audiometry applications is interesting and valuable for future research or development through primary studies. Numerous previously conducted primary studies confirm this. A previous systematic review found ten studies discussing the validation of five audiometric applications worldwide (6). As part of this systematic review, the authors found several studies that tested the validity of smartphone-based PTA applications. In this review, we searched for available applications in Indonesia, and 17 publications discussed six smartphone-based PTA applications. The 17 publications were downloaded and reviewed to verify the six smartphone-based audiometry applications by comparing them with the conventional PTA, the gold standard for evaluating hearing function (Table 1) (7,14-29). 

Most studies have revealed that smartphone-based audiometry is reliable for screening hearing loss. Several factors strongly influence the advantages and disadvantages of audiometry application in assessing hearing function. These factors could not be adequately controlled based on the standard conventional audiometry. These factors include test environment noise (background noise), transducer type and method of use, calibration implementation, the digital-to-analog converter of the device, the ability to operate a smartphone, the user’s attention when testing the application, the presence of cerumen in the ear canal, and user mistakes in application testing (7,14-24,28,29).

The noise of the test environment (background noise) is a very influential factor constantly mentioned in the literature affecting the results of smartphone-based audiometric hearing function testing. The higher the environmental noise level, the worse the test results or the results of the test person’s hearing threshold. It will increase the application’s sensitivity while decreasing its specificity in detecting hearing loss. When performed in a soundproof booth, the application will produce a hearing threshold close to the threshold obtained with conventional audiometry; however, the room cannot be used in everyday life for independent use. Nonetheless, the room adjustment to the noise of the test environment (background noise) should be kept to a minimum (6,7,15,16,19,20,22,23).

The type of transducer and how to use a suitable transducer to minimize environmental noise entering the participant’s ear are other factors affecting the application validity and are still related to environmental noise. Standard audiometric headphones are used in conventional audiometry. In contrast, most iOS application validation studies used Apple earbuds in their research because of their compatibility with iOS smartphone devices and ease of availability (7,15,16,19,21,23). Earbuds are theoretically recommended as an effective way to reduce background noise and placed in the ear canal to provide approximately 30–40 dB of noise attenuation. The placement of the earbuds is also crucial because it affects the accuracy of the measurement results, especially at low frequencies. Earphones/earbuds and circumaural earmuffs are ideally used while monitoring the test noise level. Although this is ideal, it contradicts the screening principle of using an application with minimal hardware to ensure the ease of accessibility of the smartphone-based audiometry application (7).

In addition to considering environmental noise and transducers when using smartphone-based audiometry, the application and tool calibration should be implemented. Calibration is an essential step in any testing procedure. It helps ensure equipment output and varies smartphone and transducer combinations. 

A certified electroacoustic laboratory continuously calibrates conventional audiometry before use (21). Although performing calibration is challenging in a standard procedure, simple calibration methods can be used (15,21,23,24). 

uHear^TM^ was calibrated before starting each test. Calibration is performed by asking the patients to enter a silent environment and then instructing them on the proper placement of the headphones or earbuds in the ear. The patient is then instructed to reduce the device volume to 50%. Finally, on the app, the patient selects the type of listening device to be used to complete the test (earbuds or headphones) (15). Another method is attaching the sound level meter to a headphone and setting the app’s volume to 60 dB for 1 kHz. The app was then used to adjust the headphones’ output until a 60 dB(A) reading was obtained using the sound level meter. This procedure was then repeated for each of the six audiometric frequencies used in this study. These settings were saved in the form of a calibration file (21). In contrast, the biological method involves determining the reference sound level concerning a normal hearing person’s hearing threshold. Multiple calibrations on different mobile sets of the same model allow the determination of a reliable, model-specific reference sound level (23,24).

Other factors that affect the application’s validity include the 16-bit digital-to-analog converter in the iPod/iPhone device, which limits the uHearTM application’s dynamic range to approximately 85 (15–100) dB. Because the lower dynamic range limit (0–25 dB) is within the average hearing threshold, uHear^TM^ may be less specific at lower ranges. Therefore, uHear^TM^ unsurprisingly overestimates the threshold in ears with normal hearing, particularly at lower frequencies, compared with conventional audiometry, even though the application testing is performed in a soundproof room. As a result, the uHear^TM^ application is better used in screening for impaired function, particularly in moderate to severe hearing loss (15,19). 

The results of conventional audiometry and the Audiogram Mobile^TM^ application, both tested in a soundproof room, differed. The participants were disturbed during the audiometry application test because the examiner who operated the application was also in the same soundproof booth. More distractions occurred because the participants had to pay attention to the examiner’s voice while operating the iPad. This is certainly different from the test setup used for conventional audiometers, in which the examiner is outside the soundproof booth. Nonocclusive cerumen can change the acoustics of the ear canal and the overall sound pressure in the ear canal at various high frequencies. Because smartphone-based audiometric application testing should be performed independently, users should ensure that the ear canal is clean before the test (21). 

User error is another factor that affects the validity of an audiometric application while testing the application. Users can direct it to any result they want because, unlike conventional audiometry, audiologists perform the test administration (14). Using audiometry applications supervised by audiologists is preferred. This ensures no user errors, such as eliminating specific testing frequencies, can occur by accidentally pressing buttons or switching sides of the headphones and monitoring environmental noise (24). Users must understand the application instructions before avoiding user errors when operating the application (20).

Another factor affecting the validity of smartphone-based audiometry is that the older population may be unable to operate a smartphone because they are not actively using one. This factor contributes to poor test results because the older population is frequently overlooked when tapping the screen (16-18). Conventional audiometry is the gold standard of hearing testing performed by audiologists in appropriate facilities. Because it is not available in every primary health facility because of financial constraints, most patients are referred to better health facilities for diagnostic audiometry examinations by primary health doctors (19,22). This makes it challenging to evaluate many people using conventional audiometry. To overcome this, a new hearing function assessment method, smartphone-based audiometry, has been widely developed as a hearing function screening method to provide patients with easy access and reliable results. The low cost of testing with smartphone-based audiometry and its simplicity and ease of use make it preferable to conventional audiometry testing, even though it is only limited to screening. Smartphone-based screening has great potential due to the use of smartphones and cellular networks globally (14,29). 

Most of the literature in this review reported that smartphone-based audiometry was still inferior to conventional audiometry for screening hearing loss. This difference was found to be due to many factors affecting the test results in smartphone-based audiometry applications, which are difficult to control properly. The influencing factors include the noise of the test environment (background noise), the type and method of the transducer, the implementation of calibration, the digital-to-analog converter of the device, the ability to operate a smartphone, the user’s attention when testing the application, the presence of cerumen in the ear canal, and user errors in testing. Furthermore, all reviewed studies in this systematic review demonstrate that smartphone-based audiometry is not intended to detect hearing loss more precisely or specifically. Because smartphone-based audiometry applications only test the air conduction hearing threshold using a transducer in the form of paired headphones to cover the outer ear, determining whether a person has conductive, sensorineural, or mixed hearing loss is impossible (7,14-29). 

The cut-off threshold used to assess a person experiencing hearing loss in almost all smartphone-based audiometric validation literature differs from the conventional PTA cut-off threshold under the standards and considerations of ENT specialists (7,14-29), who use a cut-off threshold of >40 dB, implying that this tool is intended for screening people with moderate or worse hearing loss (7,15-18,20,27). As a result, although this smartphone-based audiometry application cannot be used as a diagnostic tool for assessing hearing function, it can be used as a hearing function screening tool in areas where conventional audiometry is difficult to access. Using this screening method, patients with poor hearing function assessment results can be immediately referred to a better health facility (conventional PTA is available) for further evaluation and treatment by an ENT specialist to improve the patient’s quality of life (6,7,14-29).

## Conclusion

Smartphone-based audiometry can be used as a screening modality or an alternative to assess hearing function. uHear^TM^, Audiogram Mobile^TM^, AudCal^TM^, Hearing Test^TM^ e-audiologia, and Wulira^TM^ applications that have been validated or have good validity are recommended for use in areas without access to conventional audiometric examinations, primary health services, and independent use in Indonesia. Smartphone-based audiometry is inexpensive, simple, and more accessible than conventional audiometric testing. Smartphone-based audiometry cannot replace conventional audiometry in assessing hearing function because it can only measure the air conduction threshold and thus cannot determine hearing loss. Furthermore, its validity is not as good as that of conventional audiometry. Several factors that influence the measurement results, such as conventional audiometry, are uncontrollable.
